# Life Stressors and Depressive Symptoms: The Moderating Roles of Alcohol Consumption and Age

**DOI:** 10.3390/brainsci15101126

**Published:** 2025-10-21

**Authors:** Jiwan Moon, Sang-Won Jeon, Yoosuk An, Sung Joon Cho

**Affiliations:** 1 Department of Psychiatry, Kangbuk Samsung Hospital, Sungkyunkwan University School of Medicine, Seoul 03181, Republic of Korea; jiwan4524@gmail.com (J.M.); sangwonyda@hanmail.net (S.-W.J.); 2 Workplace Mental Health Institute, Kangbuk Samsung Hospital, Seoul 04514, Republic of Korea; 3 Department of Psychiatry, National Traffic Injury Rehabilitation Hospital, Yangpyeong 12564, Republic of Korea; 4 Department of Neuropsychiatry, Seoul National University Hospital, Seoul 03080, Republic of Korea; 5 Department of Psychiatry, Seoul National University College of Medicine, Seoul 03080, Republic of Korea

**Keywords:** workplace mental health, workplace stress, depressive symptoms, alcohol consumption, age, stress factors, interpersonal conflicts, traumatic events, moderation effects

## Abstract

**Background**: Mental health issues among employees are a growing concern globally, particularly in high-stress environments such as Korean workplaces. This study was conducted to analyze the relationships between life stressors and depressive symptoms among Korean employees, focusing on the moderating effects of alcohol consumption and age. **Methods**: This was a cross-sectional study of 8432 Korean employees. The participants completed assessments for depressive symptoms, alcohol consumption, and seven major life stressors (workplace stress, family relationships, interpersonal conflicts, health problems, financial strain, traumatic events, and mannerisms). Moderation analyses were performed using Hayes’ PROCESS macro. **Results**: The severity of depressive symptoms significantly increased with higher levels of alcohol consumptions. The findings indicated that age moderated the relationship between interpersonal conflict stress and depressive symptoms, suggesting that the association between interpersonal stress and depression was weaker among older individuals. Conversely, alcohol consumption moderated the relationship between traumatic event stress and depressive symptoms, suggesting that higher alcohol use intensifies the impact of traumatic stress on depression. **Conclusions**: This study provides insights into the complex interactions between life stressors, alcohol consumption, and depressive symptoms in Korean employees. The findings highlight the need for age-specific mental health interventions and early intervention for excessive alcohol consumption, especially for individuals experiencing traumatic events.

## 1. Introduction

Mental health problems among workers have been recognized as an important issue worldwide because they not only negatively affect an employee’s quality of life, but also lead to reduced productivity and increased social costs for organizations [1,2]. According to the World Health Organization [3], the global economic loss due to depression, which is strongly linked to loss of productivity, work absence, and turnover, is over USD 1 trillion per year [4,5]. Given that approximately 54% of the global working population are company workers [2], workers’ mental health problems are urgent issues at the global level.

The Korea National Health and Nutrition Examination Survey (KNHANES) [6] indicated that the overall prevalence of depression in Korea is 7.7%, and that 13.5% of workers in Korea experience depressive symptoms. This suggests that workers are more exposed to mental health problems than the general population. A substantial body of evidence indicates that exposure to a wide range of life stressors is a key driver of this increased prevalence of depressive symptoms among workers [7,8,9,10,11]. For instance, a meta-analysis by Kessler reported that work-related stress, interpersonal conflict, and financial difficulties each increase the risk of depression [7]. Given that these factors vary considerably depending on personal characteristics and environmental contexts, a clear understanding of the correlation between stress and depressive symptoms, including the factors that moderate this correlation, is essential for reducing the prevalence of depression among workers. However, previous studies have not comprehensively demonstrated how combinations of various stress-related factors affect depressive symptoms among workers [8,9,10].

Alcohol consumption is considered to have major and complex effects on the relationship between stress and depression [11]. Kim et al. [12] reported that although an appropriate amount of alcohol consumption could reduce depressive symptoms, excessive alcohol consumption greatly increases the risk of depression. Alcohol consumption can contribute to short-term stress relief; however, it can also be a maladaptive coping strategy in the long term, exacerbating depression and other mental health problems [13,14]. Alcohol consumption is widely used for stress relief and social bonding in Korean workplace settings, which are characterized by high work intensity and a competitive work culture. Previous reports have indicated that Korean workers experience very high levels of work stress and exhibit poor mental health indices compared to workers in other OECD countries [15]. Exposure to chronic high-stress environments is a major risk factor for alcohol use disorder (AUD), which is closely related to ‘coping motive’ and ‘tension reduction’ mechanisms of alcohol dependency for alleviation of stress-induced psychological tension and negative emotion [16]. The combination of the unique high-stress environment in Korea with a social atmosphere of tolerance towards alcohol consumption [11,17] can increase the risk of a negative cycle of stress-related alcohol use and depressive symptoms [18].

Sociodemographic factors can affect the relationship between stress and depression. A study of Korean workers suggested that the relationship between work stress and depressive symptoms differs according to age [19]. This could be because age is an important factor that affects stress perception and coping methods [10,20,21]. The selection, optimization, and compensation theory proposed by Baltes & Baltes [22] states that stress coping ability improves with increasing age. In addition, Charles [19] reported that older adults show better emotional regulation than young adults. However, whether age has mitigating effects on different stress factors remains unclear.

Previous studies on stress and depressive symptoms were primarily focused on the relationships between single stress-related factors and depression [9]. Moreover, studies on the moderating effects of alcohol consumption on and age on various sources of stress are limited [8,23]. Therefore, the purpose of this study was to analyze the relationships between various life stressors and depressive symptoms among Korean employees. We aimed to comprehensively evaluate whether alcohol consumption and age have differential moderating effects on specific stress factors, providing a more holistic understanding of vulnerability.

The relationship between stress, alcohol, and depression is theoretically complex and can be conceptualized through different models, primarily moderation and mediation. A mediation model would propose that stress leads to increased alcohol use, which in turn leads to depression (i.e., alcohol use as a mechanism). In contrast, a moderation model, which is the focus of the present study, seeks to identify whether a third variable (e.g., alcohol use) changes the strength of the relationship between stress and depression. While both models are plausible, our primary research question was to identify factors that characterize vulnerable subgroups, making a moderation analysis the most direct approach for our objective.

## 2. Materials and Methods

### 2.1. Study Design and Participants

This was a retrospective, cross-sectional study conducted using anonymized data obtained from employees aged 19–65 years who underwent workplace mental health screening at the Workplace Mental Health Institute of Kangbuk Samsung Hospital (Seoul, Republic of Korea). The study was conducted between April 2020 and November 2022. Of 12,565 persons who completed the study questionnaires, 8432 from 21 organizations who provided all the sociodemographic information required for analyses were included in this study.

All the study procedures were approved by the institutional review board at Kangbuk Samsung Hospital (KBSMC 2022-03-046). As this study was conducted using anonymized data collected through regular mental health screening, the board waived the need for participant consent. This study was conducted in adherence with the guidelines of the Declaration of Helsinki.

### 2.2. Assessments

The participants’ sociodemographic data, including sex, age, education level, marital status, average weekly work duration in the last year, and monthly wage in the last year, were collected for analysis.

The participants’ depressive symptoms were assessed using the Korean version of the Center for Epidemiological Studies Depression (CES-D) scale. The CES-D is a self-report scale that consists of 20 items, with a total score of 60 points. Higher scores indicate more severe depressive symptoms [24,25]. The internal consistency of the Korean version of the CES-D has been demonstrated previously (Cronbach’s α = 0.977) [26].

To evaluate sources of the stress experienced by employees, the participants were shown a list of major life stressors, including workplace stress, family relationships, interpersonal conflicts, health problems, financial strain, traumatic events, and mannerisms, and asked to choose those that had caused them stress in the last month. For each stressor selected, participants then rated the extent to which it affected their daily activities on a scale from 0 (no impairment whatsoever) to 100 (severe impairment). For the analysis, if a participant did not select a stressor, they were assigned a score of 0 for that variable. Thus, a score of 0 on any stressor variable indicates the absence of that stressor or the absence of impairment from it in the past month. The above-mentioned seven stress factors were selected based on the stress questionnaire used in the KNHANES [27], which was based on the Global Assessment of Recent Stress Scale [28].

The severity of alcohol dependence was evaluated using the Korean version of the Alcohol Use Disorder Identification Test (AUDIT-K) [29]. The AUDIT is used in screening for AUD and consists of 10 items based on three subfactors: amount and frequency of drinking, drinking-related problems, and alcohol dependence. Items 1 to 8 are rated on a 5-point scale from 0 to 4 points, whereas items 9 to 10 are rated on a 3-point scale of 0, 2, or 4 points. Higher total scores indicate a greater risk of AUD. For males, scores of 0–9 points are categorized as low-risk drinking, 10–19 points as hazardous drinking, and 20–40 points as possible alcohol dependence. For females, scores of 0–5 points are categorized as low-risk drinking, 6–9 points as hazardous drinking, and 10–40 points as possible alcohol dependence. The internal consistency of the AUDIT-K has been demonstrated to be satisfactory (Cronbach’s α = 0.82) [30].

### 2.3. Statistical Analysis

The sociodemographic and clinical characteristics of the participants were presented as means and standard deviations (SDs) or frequencies. Regarding alcohol consumption, the participants were divided into low-risk drinking, hazardous drinking, and possible alcohol dependence groups. Welch’s ANOVA was conducted to analyze the differences in CES-D scores between these groups when the assumption of homogeneity of variances (Levene’s test) was not satisfied. For post hoc analysis, Dunnett’s T3 test was used.

Model 1 of the Statistical Package for the Social Sciences (SPSS) PROCESS Macro version 4.3.1, developed by Hayes [31], was used to analyze whether sociodemographic factors such as sex, age, and work duration, and AUDIT-K scores moderate the relationships between stress factors and CES-D scores. One of the stress factors was set as the independent variable in the models to analyze the moderating effects of each variable, whereas CES-D score was set as the dependent variable. Each variable of interest (sex, age, work duration, and AUDIT-K score) was treated as a moderating variable. Other sociodemographic factors and the six other stress factor scores were controlled as covariates in all analyses. Interaction effect sizes were summarized using standardized β, the incremental variance explained by the interaction block (ΔR^2^) from hierarchical linear regression with mean-centered continuous predictors, and Cohen’s *f*^2^ = ΔR^2^/(1 − R^2^ of the interaction-inclusive model); the F-change, degrees of freedom, and *p* value for the interaction block were also reported. Additionally, to facilitate interpretation, we derived standardized simple slopes for interactions that showed evidence of moderation in the primary analyses. Slopes were evaluated at moderator values of −1 SD, mean, and +1 SD using the PROCESS macro with z-standardized continuous variables, and we report 95% confidence intervals.

As a sensitivity analysis to the primary moderation testing, we conducted a global omnibus model comparison for each stressor to mitigate multiplicity and assess overall evidence of moderation. We contrasted a base model (no interactions) with a full model that simultaneously added all stressor x moderator interactions, using a likelihood-based generalized linear model. Model fit was evaluated with the likelihood-ratio test (LRT) and the Akaike/Bayesian information criteria (AIC/BIC). The LRT was computed as the difference in −2 log-likelihood, asymptotically χ^2^ with degrees of freedom equal to the number of added interaction terms (df = 28). All models adjusted for the same prespecified covariates as in the primary moderation analyses. To account for clustering within 21 organizations, we fit generalized estimating equations (GEE) models with organizations as clusters, using robust standard errors and Wald tests, while retaining the same fixed-effect terms as in the primary models.

All statistical analyses were performed using the SPSS version 23.0 for Windows (IBM Corp., Armonk, NY, USA). Statistical significance was defined as *p* < 0.05.

## 3. Results

### 3.1. Sociodemographic and Clinical Characteristics of the Participants

The sociodemographic and clinical characteristics of the participants are shown in Table 1. The proportion of male participants in the study was higher than that of the female participants (58.8% vs. 41.2%). The mean age of the participants was 37.2 years (SD, 8.8), their mean CES-D score was 16.0 (SD, 9.9), and their mean AUDIT-K score was 8.1 (SD, 6.7).

### 3.2. Preliminary Analysis: Differences in Depressive Symptom Severity Across Stages of Alcohol Use

The mean CES-D scores of the drinking groups are as follows: 15.1 (SD, 9.8) for the low-risk drinking group (*n* = 4675), 15.9 (SD, 9.5) for the hazardous drinking group (*n* = 2517), and 19.6 (SD, 10.5) for the possible alcohol dependence group (*n* = 1240). Levene’s test indicated that the assumption of homogeneity of variances was not satisfied (*p* < 0.001); therefore, Welch’s ANOVA was performed. The differences in mean CES-D scores between the three groups were statistically significant (*p* < 0.001). Dunnett’s T3 test was used for post hoc analysis, and significant differences between the low-risk drinking group and the hazardous drinking group (*p* = 0.008), the hazardous drinking group and the possible alcohol dependence group (*p* < 0.001), and the low-risk drinking group and the possible alcohol dependence group (*p* < 0.001) were observed.

### 3.3. Analysis of Moderating Effects

#### 3.3.1. Sociodemographic Factors

**Figure 1:**
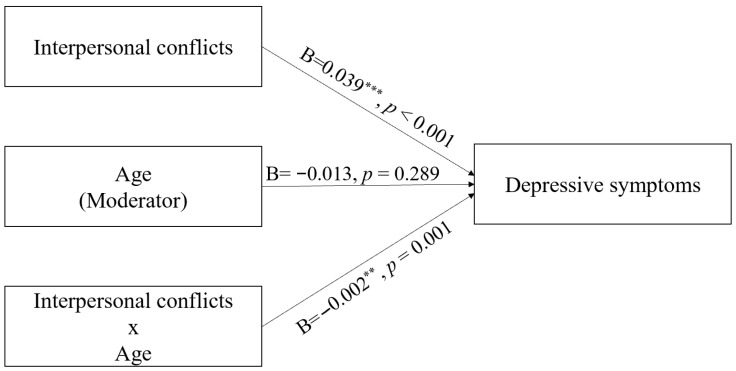
Moderating effect of age on the relationship between interpersonal conflict stress and depressive symptoms. Paths display unstandardized coefficients (B). Interpretation is based on standardized effect sizes and 95% CIs; *p*-values in the figure are provided for reference. Full estimates are provided in Table 2 and its footnote. *p* < 0.05, ** *p* < 0.01, *** *p* < 0.001.

Among the sociodemographic factors analyzed, age showed a moderating effect on the relationships between interpersonal conflict stress (among the seven life stress factors assessed) and total CES-D score (standardized β = −0.028; 95% CI [−0.044, −0.012]; see Table 2 footnote for ΔR^2^ and Cohen’s f^2^). Specifically, the effect of interpersonal conflict stress on depressive symptoms decreased with increasing age. Standardized simple-slope analyses further quantified this pattern: at −1 SD of age, β = 0.097 (95% CI [0.074, 0.119]); at the mean, β = 0.068 (95% CI [0.050, 0.087]); and at +1 SD, β = 0.040 (95% CI [0.013, 0.067]). These findings indicate that younger employees are more vulnerable to depressive symptoms in the context of interpersonal conflict than older employees. Figure 1 and Table 2 show the results of the analysis of the moderating effects of age. No other life stress factors showed evidence of moderation by age. In addition, of the potential sociodemographic moderators examined (sex, age, and work duration), only age showed a significant moderating effect. Sex and work duration did not show significant moderating effects on any of the stress factors.

#### 3.3.2. AUDIT-K Score

**Figure 2:**
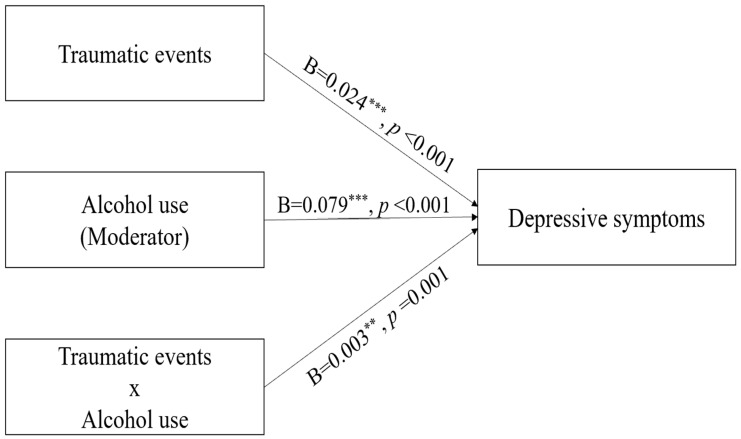
Moderating effect of alcohol use on the relationship between traumatic events and depressive symptoms. Paths display unstandardized coefficients (B). Interpretation is based on standardized effect sizes and 95% CIs; *p*-values in the figure are provided for reference. Full estimates are provided in Table 3 and its footnote. *p* < 0.05, ** *p* < 0.01, *** *p* < 0.001.

The traumatic events × AUDIT-K interaction was positive (standardized β = 0.028; 95% CI [0.012, 0.044]; see Table 3 footnote for ΔR^2^ and Cohen’s f^2^). Specifically, higher alcohol consumption was associated with a stronger effect of stress due to traumatic events on depressive symptoms. Standardized simple-slope analyses indicated that the association between traumatic events and CES-D score was β = 0.005 (95% CI [−0.017, 0.027]) at −1 SD of AUDIT-K, β = 0.030 (95% CI [0.013, 0.046]) at the mean, and β = 0.054 (95% CI [0.033, 0.076]) at +1 SD. These results suggest that participants with high AUDIT-K scores showed a stronger correlation between traumatic stress and depressive symptoms than those with low AUDIT-K scores. Figure 2 and Table 3 show the results of the analysis of the moderating effects of AUDIT-K score. No other life stress factors showed evidence of moderation by AUDIT-K score.

### 3.4. Sensitivity Analysis

#### 3.4.1. Global Omnibus Model Comparison

In the global omnibus comparison, the full model showed improved fit over the base model (LRT: χ^2^ (28) = 96.38, *p* < 0.001), with a decrease in AIC (ΔAIC = −40.38). By contrast, BIC increased (ΔBIC = +156.73). These results align with the primary moderation findings under the LRT/AIC criteria, although BIC did not favor the full model, suggesting conservative evidence.

#### 3.4.2. Clustering by Organization (GEE)

Using GEE with organizations as clusters, the age × interpersonal conflicts interaction was negative (B = −0.002, 95% CI [−0.003, −0.001]; robust SE = 0.0004, Wald χ^2^ = 18.03, df = 1, *p* < 0.001), and the traumatic events × AUDIT-K interaction was positive (B = 0.003, 95% CI [0.000, 0.006]; robust SE = 0.0014, Wald χ^2^ = 4.16, df = 1, *p* = 0.041). Coefficient estimates were similar in magnitude and direction to those from the primary models.

## 4. Discussion

This study’s primary contribution lies in its comprehensive analysis of how age and alcohol consumption differentially moderate the relationship between seven distinct life stressors and depressive symptoms in a large cohort of Korean employees. By moving beyond single-stressor models, our findings offer a more nuanced understanding of how specific vulnerabilities intersect with specific stressors, a valuable step for developing targeted mental health interventions in the workplace. Our analyses consistently showed a positive association between alcohol consumption and depressive symptoms. The main effect of the continuous AUDIT-K score was significant in our moderation models, and a preliminary descriptive analysis also showed that mean depressive symptom scores increased from the low-risk drinking group to the possible alcohol dependence group. This finding is consistent with the results of previous studies [32]. In addition, the increased severity of depressive symptoms with greater alcohol consumption suggests the possibility of a dose–response relationship between alcohol consumption and depression. This finding is consistent with the well-established relationship between AUD and depression [17]. It is noteworthy that our data showed a linear dose–response relationship and did not support the “J-shaped curve” hypothesis, which suggests a potential protective effect of light drinking. This highlights the importance of early interventions for any level of problematic alcohol use among employees.

Our findings indicated that the moderating effects of alcohol use differed depending on the life stress factor. Alcohol consumption had a moderating effect on the relationship between stress from traumatic events and depressive symptoms. Specifically, a higher level of alcohol use was associated with stronger exacerbating effects of traumatic stress on depressive symptoms. This suggests that individuals who use alcohol as a coping mechanism for stress after a traumatic event could ultimately experience worsened depressive symptoms. These findings are consistent with the self-medication hypothesis of Khantzian [33]. Alcohol consumption may be used to manage painful emotions; however, it can be a maladaptive coping strategy over time [18,34], causing long-term neurobiological changes, diminishing stress resilience, and eventually worsening mental health [35,36]. These findings suggest that when designing mental health support systems for employees, it is important to identify problematic alcohol use early among those who have experienced traumatic events and to actively provide interventions for these employees. For example, alcohol use screening, programs for prevention of alcohol misuse, and psychosocial support services could be important intervention strategies for this group. Furthermore, active education and encouragement of healthier and more adaptive coping strategies for stress management without dependence on alcohol are essential.

In this study, age showed a moderating effect on the relationship between interpersonal conflict stress and depressive symptoms. Specifically, increased age mitigated the effects of interpersonal stress on depressive symptoms. This supports the socioemotional selectivity theory of Carstensen et al. [37]. This theory states that aging is accompanied by improved ability to control emotions, development of stress coping strategies, and enhanced ability to handle complex social situations, which could act as a buffer to psychological effects, such as interpersonal conflicts. Considering the effects of age observed in the present study, an age-based approach is needed in the development of workplace mental health programs and screening of potential participants. For example, for young employees, screening and intervention strategies that place greater weight on interpersonal stress scores could be helpful in identifying individuals at risk of developing depressive symptoms. In addition, personalized programs that focus on training in communication skills, conflict resolution, and stress management could be especially effective. As a sensitivity analysis, a global omnibus comparison was directionally consistent with the primary moderation findings under the LRT/AIC criteria. Accounting for organizational clustering via GEE did not alter the substantive interpretation. Nevertheless, effect sizes were small and BIC did not favor the full model. Accordingly, we infer that moderation is present but small in magnitude, and we interpret the findings with appropriate caution given the large sample size.

One of the strengths of this study is its large sample size (*n* = 8432), which increased the statistical reliability and generalizability of the results. Given that the sample size of the present study is larger than those of many previous studies [38,39,40], our findings can contribute to a broader understanding of stress and mental health problems among workers in Korea and worldwide. In addition, the present study is valuable because we conducted a more integrative analysis by comprehensively evaluating the relationships between seven major life stress factors, alcohol consumption, depressive symptoms, and sociodemographic factors. By demonstrating that alcohol consumption and age exert different moderating effects depending on the stress factor, we highlighted the need for personalized approaches to mental health interventions for employees.

This study has some limitations that should be acknowledged. Firstly, the cross-sectional design made it difficult to determine causal relationships. This design also prevents us from formally testing plausible alternative models, such as a mediational model where alcohol use serves as a coping mechanism linking stress to depression. Future longitudinal studies are essential to disentangle these complex temporal relationships and formally test both moderation and mediation pathways. Secondly, our sample was drawn from employees participating in a workplace mental health screening program, largely from major corporations. Therefore, the findings may not be fully generalizable to employees in small-to-medium-sized enterprises or other specific industries. Thirdly, all measures were based on self-report, which is susceptible to social desirability and recall bias. Future studies could provide a more robust understanding by incorporating objective measures, such as company-level data on absenteeism or physiological stress indicators (e.g., salivary cortisol). Fourthly, we were unable to account for other potential moderating variables that could affect the relationship between stress and depressive symptoms, such as social support, personality trait, and organizational culture. Inclusion of these variables in future research will allow for a more systematic understanding of the stress–depression relationship. Fifthly, while the life stressor items were based on established instruments used in a national survey (KNHANES), specific reliability and validity data for the seven-item scale as used in our screening were not formally established. Future research could benefit from using more comprehensively validated multi-item scales for each stress domain. Sixthly, due to the secondary nature of the anonymized dataset, we did not have access to item-level data, which prevented the calculation of internal consistency coefficients (e.g., Cronbach’s alpha) for our specific sample. Instead, we have reported the reliability from large-scale Korean validation studies, which provide a strong estimate of the scales’ performance in this population.

## 5. Conclusions

In this study, we investigated the differential moderating effects of alcohol consumption and age on the relationships between specific life stress factors and depressive symptoms among Korean employees. The results indicated that the association between stress from traumatic events and depression was stronger among those with higher alcohol consumption, whereas the association between stress from interpersonal conflict and depression was weaker among older employees. These results suggest the need for multifaceted, personalized intervention strategies, including the early detection of alcohol misuse and age-specific stress interventions, for improved mental health in the workplace.

## Figures and Tables

**Table 1:** Sociodemographic and clinical characteristics of the participants (*n* = 8432).

Characteristics	
Sex, *n* (%)	
Male	4959 (58.8)
Female	3473 (41.2)
Age (years), mean ± SD	37.2 ± 8.8
Educational level (graduate), *n* (%)	
College graduate or lower	1291 (15.3)
Bachelor’s degree	5613 (66.6)
Master’s degree or higher	1528 (18.1)
Marital status, *n* (%)	
Single	3434 (40.7)
Married	4821 (57.2)
Other ^a^	176 (2.1)
Working hours (weekly, hour), mean ± SD	46.7 ± 7.7
Income (monthly), *n* (%)	
Low income	306 (3.6)
Middle income	4144 (49.2)
High income	3982 (47.2)
CES-D score, mean ± SD	16.0 ± 9.9
AUDIT-K score, mean ± SD	8.1 ± 6.7
Workplace stress, mean ± SD	35.8 ± 30.0
Family relationships, mean ± SD	15.3 ± 22.9
Interpersonal conflicts, mean ± SD	10.0 ± 18.2
Health problems, mean ± SD	16.2 ± 21.9
Financial strains, mean ± SD	17.9 ± 23.7
Traumatic events, mean ± SD	3.4 ± 12.3
Mannerisms, mean ± SD	22.0 ± 25.5

Abbreviations: SD—standard deviation; CES-D—Center for Epidemiological Studies Depression; AUDIT-K—Korean version of the Alcohol Use Disorder Identification Test. ^a^ includes separated, divorced, widowed.

**Table 2:** Moderating effect of age on the relationship between interpersonal conflict stress and depressive symptoms (*n* = 8432).

Predictor	B	SE	*t*	*p*	LLCI	ULCI
Interpersonal conflicts	0.039	0.005	7.498 ***	<0.001	0.029	0.049
Age	−0.013	0.013	−1.060	0.289	−0.038	0.011
Interpersonal conflicts x age ^†^	−0.002	0.001	−3.364 **	0.001	−0.003	−0.001
Covariates:						
Sex (reference: male)	0.709	0.175	4.062 ***	<0.001	0.367	1.052
Educational level (reference: bachelor’s degree)						
College graduate or lower	0.836	0.236	3.545 ***	<0.001	0.374	1.299
Master’s degree or higher	−0.449	0.225	−1.995 *	0.046	−0.889	−0.008
Marital status (reference: single)						
Married	−1.289	0.217	−5.950 ***	<0.001	−1.714	−0.864
Other ^a^	0.968	0.610	1.585	0.113	−0.229	2.164
Working hours (weekly, hour)	0.036	0.011	3.273 **	0.001	0.015	0.058
Income (reference: middle income)						
Low income	1.132	0.449	2.521 *	0.012	0.252	2.013
High income	−0.773	0.188	−4.099 ***	<0.001	−1.142	−0.403
Life stressors (covariates):						
Workplace stress	0.134	0.003	42.741 ***	<0.001	0.128	0.140
Family relationships	0.054	0.004	13.363 ***	<0.001	0.046	0.062
Health problems	0.022	0.004	5.083 ***	<0.001	0.013	0.030
Financial strains	0.017	0.004	4.304 ***	<0.001	0.009	0.025
Traumatic events	0.026	0.007	3.733 ***	<0.001	0.012	0.039
Mannerisms	0.074	0.004	20.536 ***	<0.001	0.067	0.081
F = 374.117 ***, R2 = 0.430, R2 change = 0.001 **						

^†^ Effect size for the interaction: β = −0.028; ΔR^2^ = 0.001; Cohen’s f^2^ = 0.002. F-change = 11.31; df = 1, 8413; *p* = 0.001. Abbreviations: B—unstandardized coefficient; β—standardized coefficient; SE—standard error; LLCI—lower limit confidence interval; ULCI—upper limit confidence interval. ^a^ includes separated, divorced, widowed. * *p* < 0.05, ** *p* < 0.01, *** *p* < 0.001.

**Table 3:** Moderating effect of alcohol use on the relationship between traumatic events and depressive symptoms (*n* = 8432).

Predictor	B	SE	*t*	*p*	LLCI	ULCI
Traumatic events	0.024	0.007	3.495 ***	<0.001	0.011	0.037
Alcohol use	0.079	0.013	6.134 ***	<0.001	0.054	0.105
Traumatic events x alcohol use ^‡^	0.003	0.001	3.444 ***	0.001	0.001	0.005
Covariates:						
Sex (reference: male)	1.018	0.180	5.640 ***	<0.001	0.664	1.371
Age	−0.006	0.013	−0.454	0.650	−0.030	0.019
Educational level (reference: bachelor’s degree)						
College graduate or lower	0.842	0.235	3.575 ***	<0.001	0.380	1.303
Master’s degree or higher	−0.378	0.225	−1.685	0.092	−0.819	0.062
Marital status (reference: single)						
Married	−1.258	0.216	−5.815 ***	<0.001	−1.682	−0.834
Other ^a^	0.856	0.608	1.408	0.159	−0.336	2.047
Working hours (weekly, hour)	0.035	0.011	3.162 **	0.002	0.013	0.056
Income (reference: middle income)						
Low income	1.172	0.448	2.616 **	0.009	0.294	2.051
High income	−0.864	0.189	−4.582 ***	<0.001	−1.234	−0.494
Life stressors (covariates):						
Workplace stress	0.133	0.003	42.541	<0.001	0.127	0.139
Family relationships	0.054	0.004	13.235	<0.001	0.046	0.062
Interpersonal conflicts	0.040	0.005	7.963	<0.001	0.030	0.050
Health problems	0.021	0.004	5.042	<0.001	0.013	0.030
Financial strains	0.016	0.004	3.891	<0.001	0.008	0.023
Mannerisms	0.074	0.004	20.352	<0.001	0.066	0.081
*F* = 357.212 ***, R^2^ = 0.433, R^2^ change = 0.001 **						

^‡^ Effect size for the interaction: β = 0.028; ΔR^2^ = 0.001; Cohen’s f^2^ = 0.002. F-change = 11.87; df = 1, 8413; *p* = 0.001. Abbreviations: B—unstandardized coefficient; β—standardized coefficient; SE—standard error; LLCI—lower limit confidence interval; ULCI—upper limit confidence interval. ^a^ includes separated, divorced, widowed. ** *p* < 0.01, *** *p* < 0.001.

## Data Availability

The data that support the findings of this study are available from the corresponding authors upon reasonable request.

## Abbreviations

The following abbreviations are used in this manuscript:

AUD	Alcohol use disorder
ANOVA	Analysis of variance
CES-D	Center for Epidemiological Studies Depression
AUDIT-K	Korean version of the Alcohol Use Disorder Identification Test
